# Giant left main coronary artery aneurysm in a young adult female with suspected incomplete Kawasaki disease: a case report

**DOI:** 10.3389/fmed.2026.1916098

**Published:** 2026-08-10

**Authors:** Lei Zhao, Ruibin Li, Yi Zhang, Jidong Zhang

**Affiliations:** Department of Cardiology, The Second Hospital of Hebei Medical University, Shijiazhuang, China

**Keywords:** coronary artery lesions, giant left main coronary artery aneurysm, incomplete Kawasaki disease, late sequelae, young adult female

## Abstract

**Background:**

Kawasaki disease (KD) is a systemic vasculitis predominantly occurring in pediatric populations. Giant coronary artery aneurysm (gCAA) involving the left main coronary artery (LMCA) is an extremely rare long-term complication of KD. Adult-onset or late-diagnosed KD-related giant LMCA aneurysm in young female patients represents a rare clinical entity with high risks of intracoronary thrombosis, myocardial infarction, and sudden cardiac death. Due to absent classic childhood KD symptoms, such aneurysms are commonly misdiagnosed as atherosclerotic lesions or congenital coronary malformations upon first admission. Notably, no gold-standard laboratory test exists to confirm remote childhood KD; diagnoses in adult survivors rely heavily on exhaustive exclusion of alternative vasculopathies combined with pathognomonic coronary angiographic morphology, rather than angiographic findings alone.

**Case presentation:**

This study reports a case of a 35-year-old previously healthy female with a one-month history of intermittent exertional chest pain. She denied classic childhood manifestations of KD and had no conventional cardiovascular risk factors. Invasive coronary angiography confirmed a giant fusiform LMCA aneurysm with a mean diameter of 12.78 mm, accompanied by mild mural calcification and reduced intracavitary perfusion. Typical beaded saccular and fusiform aneurysms were also observed in the left anterior descending and right coronary arteries (RCA). Laboratory tests excluded autoimmune vasculitis,infectious arteriopathy and atherosclerotic heart disease. Combined with highly characteristic angiographic coronary vasculopathy patterns and complete exclusion of competing diagnoses, we established a presumptive diagnosis of chronic vascular sequelae derived from unrecognized incomplete KD, rather than a definitive confirmed diagnosis. The patient received lifelong dual antiplatelet plus anticoagulant therapy, and underwent drug-coated balloon angioplasty for RCA lesions. No early major adverse cardiovascular events were documented within the 6-month structured short-term follow-up; long-term thrombotic risk remains persistent and requires prolonged serial surveillance.

**Conclusion:**

Unrecognized incomplete KD in childhood may progress to symptomatic giant LMCA aneurysms in young adulthood, even without typical acute KD manifestations. For young female patients with idiopathic isolated giant LMCA aneurysms, antecedent occult incomplete KD should be highly suspected. Long-term individualized antithrombotic therapy and close imaging surveillance are essential to prevent life-threatening cardiovascular complications.

## 1 Introduction

Kawasaki disease (KD) is an acute, self-limiting systemic vasculitis that predominantly affects children <5 years old (1). Coronary artery lesions constitute the most severe complication of KD and serve as the primary predictor of long-term adverse cardiovascular prognosis. gCAA is defined as coronary dilatation with an internal diameter≥8 mm or twice the diameter of the adjacent normal vessel, which represents a devastating adverse outcome of KD occurring in less than 1% of pediatric patients treated with standard intravenous immunoglobulin (IVIG) (2). Per the 2024 AHA KD scientific statement, giant coronary aneurysm severity grading should incorporate body surface area (BSA)-adjusted coronary z-scores to avoid misclassification in patients with small body size, rather than relying solely on absolute luminal diameter cutoffs (1). This guideline also systematically describes the characteristic multifocal, beaded fusiform and saccular coronary ectasia pathognomonic for remote KD injury, a hallmark feature that distinguishes KD-related aneurysms from lesions induced by atherosclerosis, vasculitis or congenital malformations, consistent with the classic morphological criteria proposed by Gordon et al. (3) in their 2009 JACC review. Most KD-associated coronary aneurysms are detected and managed during the acute pediatric stage, mainly affecting the LAD artery and RCA. Isolated giant aneurysm of the LMCA secondary to KD is extremely rare, especially among young adult females without confirmed childhood KD history (4). Atherosclerotic lesions and congenital coronary malformations are the leading causes of adult-onset coronary aneurysms, whereas undiagnosed incomplete KD is commonly neglected during differential diagnosis for unexplained coronary dilatation in young adults. A critical differential diagnosis frequently overlooked in young female patients with diffuse multivessel beaded coronary aneurysms is burnt-out Takayasu arteritis (TA), a large-vessel vasculitis that may present with normal acute-phase inflammatory markers during its chronic quiescent phase; comprehensive thoracic aorta and supra-aortic branch vessel imaging is mandatory to rule out this entity. Of note, giant LMCA aneurysms confer markedly elevated risks of intracoronary thrombosis, progressive stenosis, arterial dissection, vessel rupture and acute myocardial infarction, ultimately resulting in sudden cardiac death (5). Herein, we report a rare case of chronic KD sequelae presenting as a giant LMCA aneurysm in an asymptomatic young adult female, who had no classic childhood KD inflammatory manifestations and no conventional atherosclerotic risk factors. This case broadens the clinical spectrum of adult residual KD vasculopathy and highlights the necessity of screening for occult incomplete KD in young adults diagnosed with idiopathic gCAA. We further emphasize that adult diagnoses of remote childhood incomplete KD can only be framed as presumptive/probable diagnoses, given the absence of confirmatory biomarkers and inability to retrospectively satisfy pediatric acute-phase incomplete KD scoring criteria;such diagnosis must be built on two core pillars: full exclusion of all alternative coronary aneurysm etiologies, and the presence of KD-specific coronary morphological changes, instead of depending merely on angiographic appearances.

## 2 Case presentation

### 2.1 Patient history and clinical manifestations

A 35-year-old previously healthy female presented to the cardiology outpatient clinic with a one-month history of intermittent exertional precordial pain and occasional palpitations. All symptoms resolved completely at rest, without associated dyspnea, syncope or peripheral edema. The patient denied all classic childhood Kawasaki disease manifestations, including prolonged high fever, polymorphous exanthema, bilateral conjunctival injection, oropharyngeal erythema, extremity swelling and cervical lymphadenopathy, and there was no documented prior diagnosis of KD. She had no medical history of hypertension, diabetes mellitus, dyslipidemia, tobacco use or alcohol intake. Her family history was unremarkable for congenital heart disease or hereditary vasculopathy. Prior to invasive angiography, non-invasive ischemia assessment was completed: treadmill stress ECG showed only mild inferior ST-segment depression without anterior wall ischemic changes; cardiac magnetic resonance (CMR) stress perfusion imaging demonstrated isolated mild reversible perfusion defects limited to the RCA territory, with no reversible ischemia in the LAD vascular bed.

### 2.2 Physical examination

All vital signs were stable: blood pressure 102/74 mmHg, heart rate 69 beats per minute, respiratory rate 18 breaths per minute, and body temperature 36.5 °C. Her height was 153 cm, weight 45 kg, with a body mass index (BMI) of 19.22. Cardiac auscultation revealed regular sinus rhythm with no murmurs, pericardial friction rubs, or gallop sounds. Pulmonary, abdominal, neurological and limb examinations yielded unremarkable results. There were no cutaneous rashes, joint swelling, mucosal ulcerations or other signs suggestive of systemic vasculitis. No asymmetric upper-extremity blood pressure gradients, carotid or subclavian bruits were detected, which are typical clinical hallmarks of Takayasu arteritis.

### 2.3 Auxiliary laboratory examinations

Routine blood counts, liver and renal function, serum lipid profile and fasting blood glucose were all within normal reference ranges. Inflammatory biomarkers were unremarkable: C-reactive protein (CRP) 2.3 mg/L and erythrocyte sedimentation rate (ESR) 12 mm/h, consistent with an absence of active systemic inflammation. Cardiac injury markers including cardiac troponin I and CK-MB, as well as N-terminal pro-brain natriuretic peptide (NT-proBNP), were normal, ruling out acute myocardial injury and heart failure. Coagulation assays revealed no hypercoagulability. Autoimmune serology, including ANA, ANCA, HLA-B27, anti-endothelial cell antibodies, antiphospholipid antibodies, antiphosphatidylserine/prothrombin IgM antibodies and lupus anticoagulant, yielded entirely negative results. Screening for infectious pathogens (*Mycoplasma pneumoniae*, Epstein–Barr virus, adenovirus) was also negative, excluding autoimmune vasculitis and acute infectious arteriopathy. Serological tests for IgG4-related disease, Behçet disease and systemic connective tissue diseases were all negative; no history of cocaine abuse, coronary injury or previous cardiac intervention was reported by the patient, which excludes these aneurysm-inducing etiologies.

### 2.4 Imaging examinations

**Figure 1:**
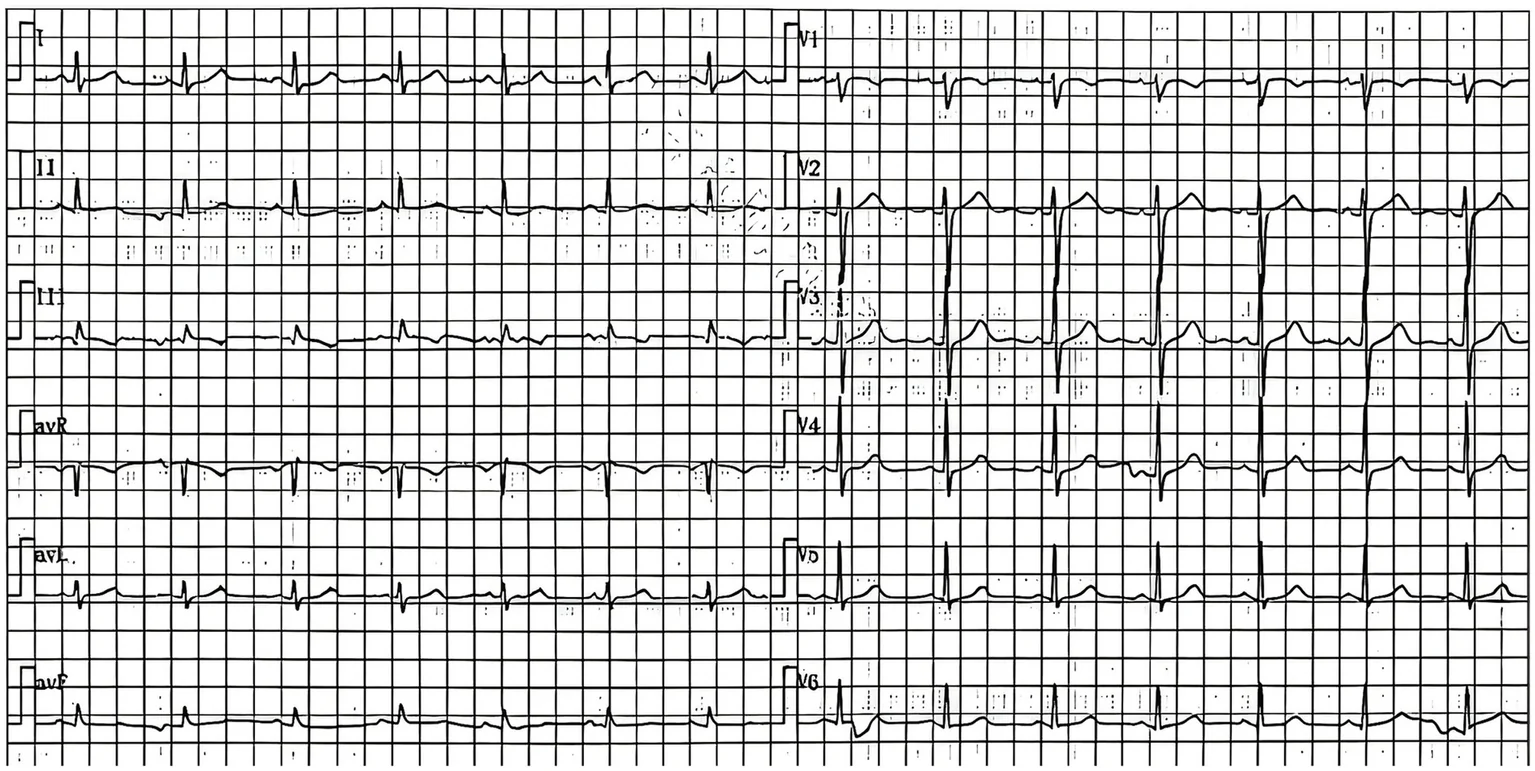
Normal sinus rhythm at 77 beats per minute, with flattened and inverted T waves in leads II, III and aVF, and no pathological Q waves.

Electrocardiography (ECG) demonstrated normal sinus rhythm, with flattened and inverted T waves in the inferior leads (II, III, aVF). No pathological Q waves were identified. (Figure 1).

Transthoracic echocardiography showed normal cardiac structure and systolic function, without pericardial effusion or valvular regurgitation. Mild dilatation of the LMCA was suspected on initial screening, and intraluminal features could not be clearly visualized.

**Figure 2:**
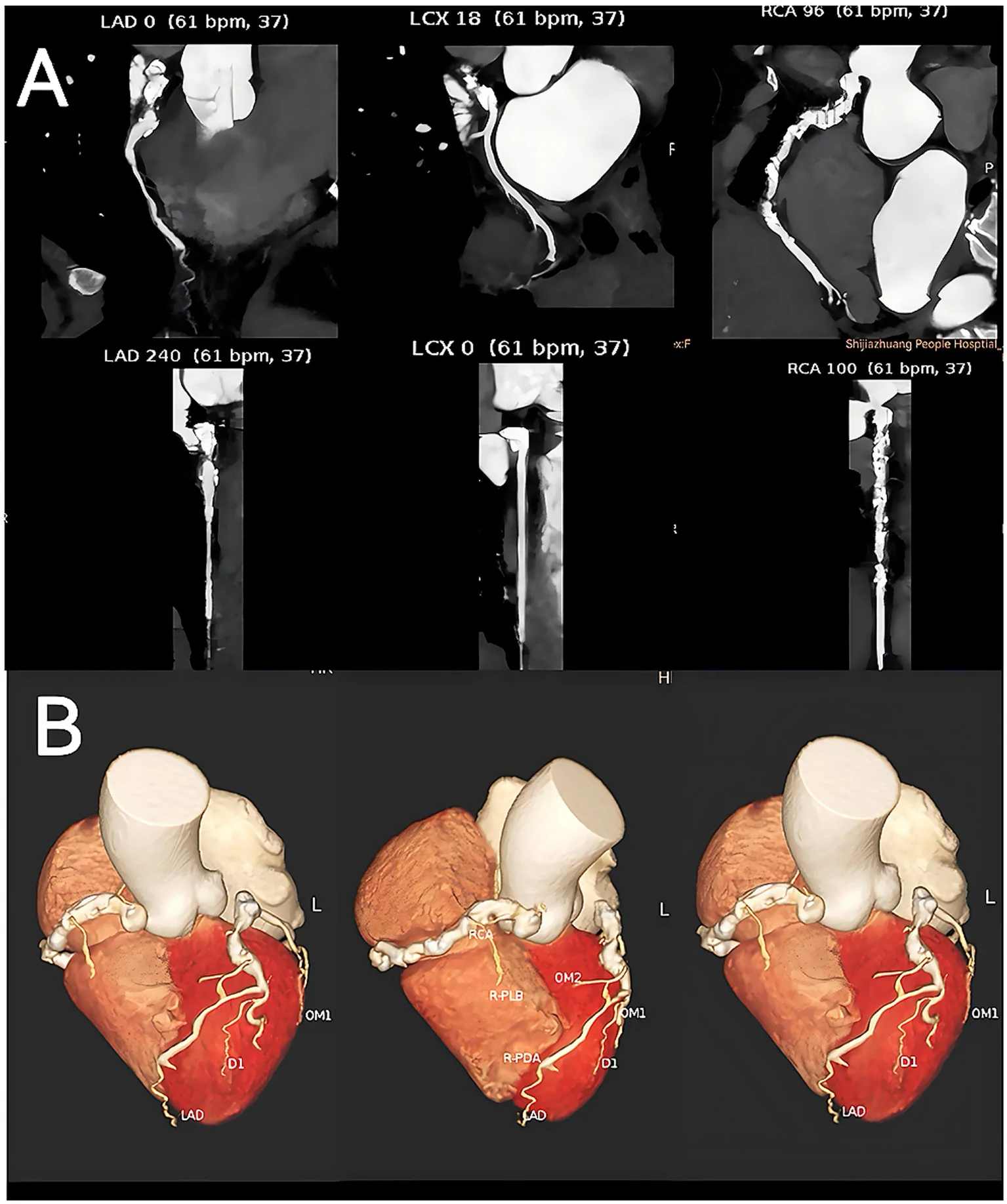
**(A)** LAD: Irregular aneurysmal ectasia with mixed plaques and severe proximal stenosis. LCX: Proximal dilatation with mural calcification; RCA: Diffuse irregular dilatation with characteristic beaded configuration and multifocal segmental stenoses, with the most severe stenosis located at the mid segment. **(B)** The LAD and RCA present multiple irregular aneurysmal ectasia with a beaded configuration.

Coronary computed tomography angiography (CCTA): complete CCTA thoracic aorta and supra-aortic branch imaging showed no circumferential vessel wall thickening, stenosis, mural edema or aortic aneurysm. CCTA revealed aneurysmal dilatation of the LMCA with mural calcified plaques. The LAD exhibited irregular aneurysmal ectasia mixed with atherosclerotic plaques, accompanied by severe stenosis at its proximal segment. The proximal left circumflex artery (LCX) showed dilatation and mural calcified plaques. The entire RCA displayed diffuse irregular dilatation with a characteristic beaded configuration. Multiple mixed plaques were distributed along the vessel wall, resulting in segmental stenoses of variable severity. The most significant stenosis is located in the mid segment of the RCA (Figure 2).

Invasive coronary angiography confirmed the isolated giant fusiform LMCA aneurysm, accompanied by sluggish intraluminal blood flow (TIMI grade 2).

**Figure 3:**
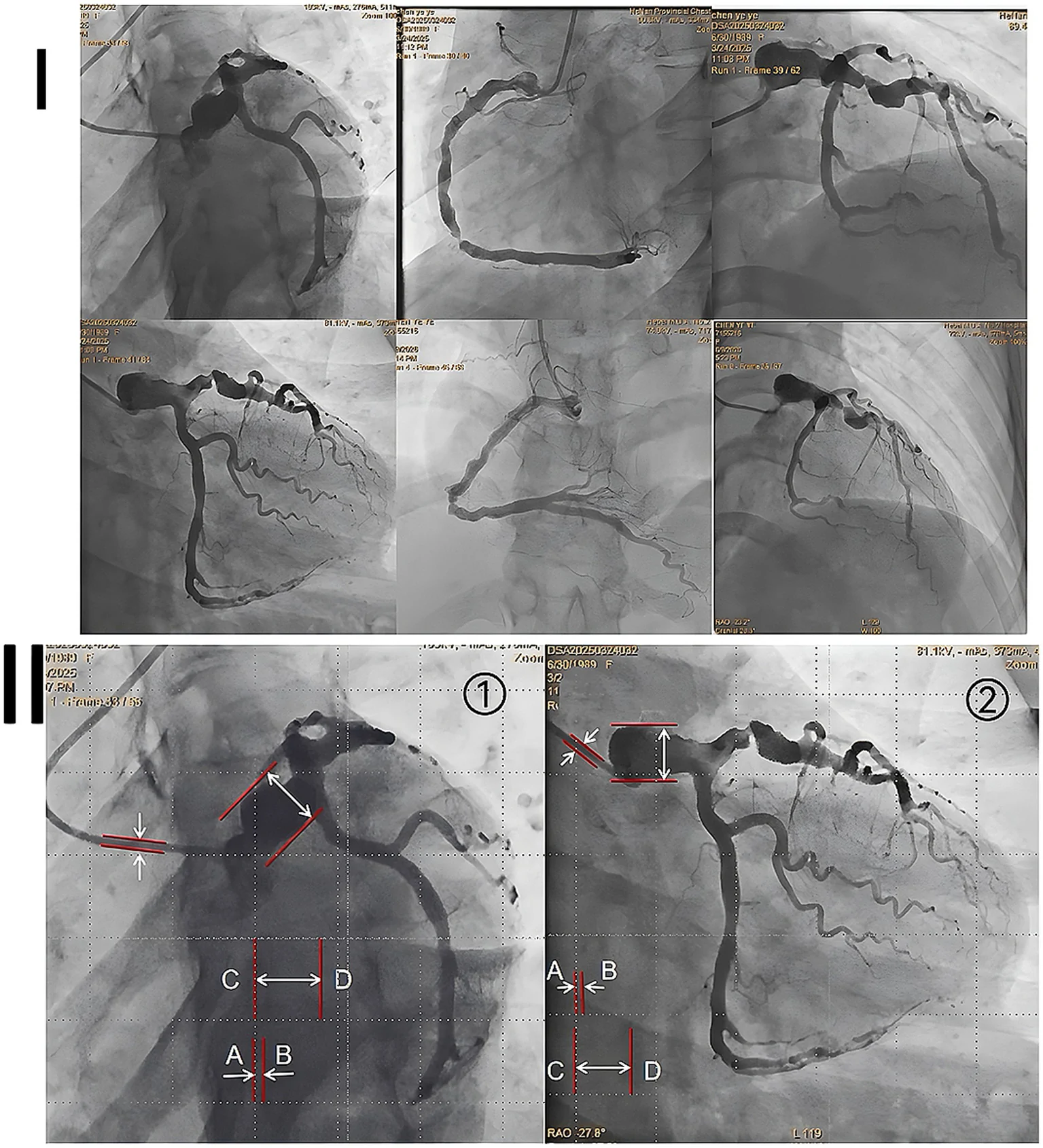
I: Invasive coronary angiography showing an isolated giant fusiform LMCA aneurysm. Proximal LAD aneurysmal dilatation with 90% focal stenosis, mild dilatation and plaques in mid/distal LAD. The LCX exhibits mural atherosclerotic plaques with mild stenosis of the mid-segment. RCA ostial aneurysmal dilatation, mild ectasia with plaques in proximal/mid RCA, and critical 95% stenosis at the second RCA bend. II: Invasive coronary angiography was performed to quantify the left main coronary artery (LMCA) aneurysm, with a 5-French diagnostic catheter (outer diameter = 1.67 mm) adopted as a radiopaque internal calibration standard. All angiographic luminal measurements were calibrated against the known catheter diameter to obtain actual vessel dimensions. ① Left anterior oblique (LAO) 27.9° plus caudal 31.7° projection. Line AB corresponds to the 5-French catheter reference with a pixel ratio of 1.3 (true diameter, 1.67 mm), and segment CD marks the maximal transverse diameter of the LMCA aneurysm (pixel ratio = 9.5). The calibrated aneurysmal diameter was calculated as 1.67 × 9.5/1.3 = 12.20 mm. ② Right anterior oblique (RAO) 27.8° plus caudal 27.9° projection. The pixel ratio of the aneurysmal lumen relative to the catheter reference was 8.0, corresponding to a calibrated diameter of 13.36 mm. The mean maximal LMCA aneurysmal diameter calculated from the two orthogonal angiographic projections was 12.78 mm.

BSA and coronary z-score quantification was performed per 2024 AHA guidelines: Patient BSA calculated via DuBois formula = 0.007184 × 153^0.725 × 45^0.425 = 1.36 m^2^. The mean maximum internal diameter of the LMCA aneurysms was 12.78 mm (Figure 3-II), with a BSA-adjusted LMCA z-score of +11.4, satisfying standardized gCAA definition (z-score ≥10 or absolute diameter ≥8 mm). Supplementary z-scores: proximal LAD aneurysm *z* = +7.8; diffuse RCA ectasia *z* = +5.3 ~ +9.1.

The proximal LAD showed aneurysmal dilatation with a focal 90% severe stenosis; mild dilatation with atherosclerotic plaques was seen along its mid and distal segments. The LCX exhibited diffuse atherosclerotic plaque burden with mild stenosis in mid segment. The RCA ostium presented aneurysmal dilatation. Its proximal and mid segments displayed mild ectasia loaded with atherosclerotic plaques, and a critical focal stenosis of approximately 95% was identified at the second bend of the RCA (Figure 3-I). Intraprocedural fractional flow reserve (FFR) testing was performed to evaluate hemodynamic significance of stenoses: RCA mid stenosis FFR = 0.66 (<0.75, hemodynamically significant); proximal LAD 90% stenosis FFR = 0.83 (>0.80, no functional ischemia).

### 2.5 Diagnostic basis

#### 2.5.1 Application of 2024 AHA incomplete KD diagnostic algorithm and scoring limitations

The 2024 AHA scientific statement provides a standardized diagnostic algorithm for incomplete KD exclusively applicable to febrile pediatric patients in the acute inflammatory phase (1), requiring 2–3 classic mucocutaneous systemic manifestations plus elevated acute-phase inflammatory biomarkers to establish a probable KD diagnosis. This patient has no verifiable history of prolonged fever or classic KD mucocutaneous lesions during childhood, and serum CRP and ESR were entirely normal at adult presentation, meaning the acute-phase incomplete KD scoring criteria cannot be retrospectively fulfilled. Given the nearly 30-year latency between presumed subclinical childhood vasculitis and adult symptomatic presentation, no validated retrospective clinical scoring tool can reliably confirm remote KD; accordingly, all diagnostic conclusions are probabilistic rather than definitive.

#### 2.5.2 Differential diagnosis of coronary aneurysms

1. Burnt-out Takayasu arteritis was prioritized as a core differential diagnosis given the patient’s young female sex and diffuse multivessel beaded coronary aneurysms, a classic TA angiographic phenotype that may present with normal ESR/CRP in chronic inactive stages. Multiple lines of evidence rule out TA: (1) No asymmetric limb blood pressure differences, large-vessel bruits, or systemic symptoms consistent with TA; (2) Complete CCTA thoracic aorta and supra-aortic branch imaging showed no circumferential vessel wall thickening, stenosis, mural edema or aortic aneurysm; (3) TA-related coronary lesions typically arise secondary to aortic root inflammatory damage, while this patient’s aneurysms are isolated to epicardial coronary arteries without aortic ostial involvement.
2. Polyarteritis nodosa: Characterized by necrotizing small-medium vessel vasculitis, typically with renal involvement, peripheral neuropathy and persistently elevated inflammatory markers; all relevant lab tests and clinical manifestations of this patient were negative, inconsistent with this diagnosis.
3. Behçet disease: No recurrent oral/genital ulcer, uveitis or cutaneous erythema nodosum; negative related autoantibodies exclude this entity.
4. Connective tissue disorders: ANA, ANCA and other autoimmune serology were all negative, without systemic multi-organ involvement.

#### 2.5.3 Core diagnostic evidence supporting presumptive incomplete KD sequelae

The patient was assigned a presumptive diagnosis of chronic vascular sequelae of unrecognized incomplete KD presenting with an isolated giant LMCA aneurysm, supported by the following evidence: (1) The patient is a young adult female with no conventional atherosclerotic risk factors; (2) The presence of an isolated giant fusiform LMCA aneurysm and diffuse multivessel beaded coronary ectasia, highly characteristic coronary vasculopathy patterns,strongly suggestive of prior undiagnosed incomplete KD; (3) Other etiologies including atherosclerosis, autoimmune vasculitis, congenital vascular malformations, infectious vasculopathies, and Takayasu arteritis were fully excluded via serology, pathogen screening and cross-sectional large-vessel imaging; (4) Laboratory testing revealed no signs of active systemic inflammation, consistent with chronic residual vascular lesions secondary to resolved KD; (5) Although the patient lacked classic clinical manifestations of acute KD in childhood,the highly characteristic coronary aneurysm morphology strongly suggested a history of subclinical incomplete KD. No serological or imaging gold standard exists to definitively confirm remote childhood KD, restricting this diagnosis to a probable, exclusion-based conclusion.

### 2.6 Treatment and follow-up

**Figure 4:**
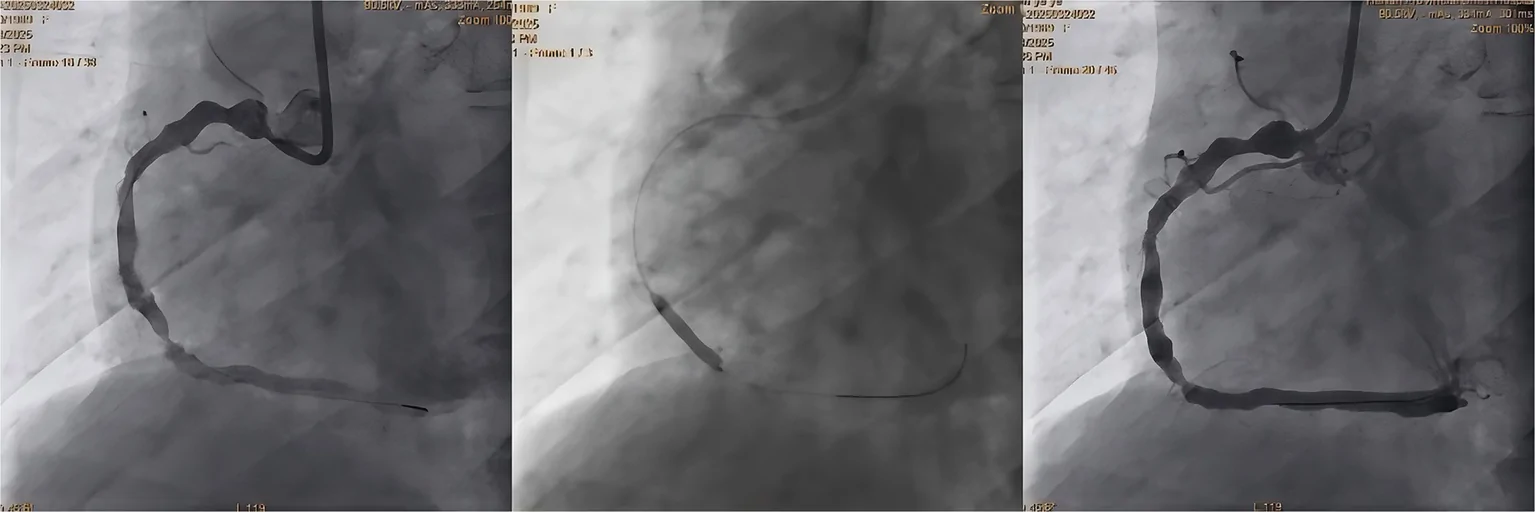
DCB angioplasty (Lepu, 3.0 × 16 mm, model: LPCDB30016) was conducted for the RCA lesion.

Based on the findings of coronary angiography and after thorough discussion of therapeutic regimens with the patient, percutaneous coronary intervention (PCI) with a drug-coated balloon (DCB, Lepu, 3.0 × 16 mm, LPCDB30016) was performed on the RCA (Figure 4). Explicit treatment decision rationale for selective revascularization: (1) The RCA mid 95% stenosis demonstrated objective inducible ischemia on stress CMR and abnormal FFR (0.66), mandating revascularization; DCB was selected over permanent metallic stents to mitigate risks of stent malapposition and late thrombosis within aneurysmal vessel segments. (2) The proximal LAD 90% angiographic stenosis was located immediately distal to the giant LMCA fusiform aneurysm, with FFR 0.83 (no hemodynamic ischemia) and no matching LAD-territory reversible perfusion defects on stress imaging. PCI at this aneurysm-stenosis transition zone carries excessive risks of coronary dissection, flow disturbance within the giant LMCA aneurysm, and heightened thrombotic complications. The heart team elected antithrombotic medical therapy and serial imaging surveillance for the functionally insignificant LAD stenosis, deferring invasive revascularization. Considering the inherently high thrombotic risk associated with giant coronary neurysms, standardized long-term antithrombotic therapy was initiated for this patient. The dual antiplatelet regimen consisted of aspirin 100 mg once daily combined with clopidogrel 75 mg once daily. Low-molecular-weight heparin was administered initially and subsequently transitioned to oral warfarin for systemic anticoagulation. Regular international normalized ratio (INR) monitoring was performed to maintain the therapeutic target range of 2.0–2.5. The patient was also counseled to implement strict lifestyle adjustments, including avoidance of intense physical exertion and adherence to sufficient rest.

At the 6-month outpatient follow-up, the patient reported no recurrent chest pain or palpitations. Serial laboratory testing revealed normal inflammatory biomarkers, myocardial enzymes, and cardiac functional parameters. Follow-up coronary angiography showed stable aneurysmal dimensions, without new mural thrombus, progressive calcification, or deterioration of intracavitary perfusion. Only short-term 6-month surveillance data are available; absence of early MACE cannot verify long-term safety or sustained efficacy of the antithrombotic regimen, as thrombotic risk of giant coronary aneurysms accumulates persistently over decades.

## 3 Discussion

Most adult residual KD coronary lesions are reported in young male patients, and giant LMCA aneurysms in young females are extremely rare. We reported a female adult patient who had no typical acute KD major manifestations in childhood, which progressed to severe giant coronary aneurysm in adulthood, further confirming that incomplete KD without obvious acute symptoms can still cause irreversible long-term vascular damage. Besides, the patient had no atherosclerosis or vasculitis-related risk factors, eliminating confounding factors and further confirming the lesion origin from KD residual vasculopathy. Isolated LMCA giant aneurysm is far less common than multi-vessel coronary aneurysms in KD, and LMCA lesions are clinically more dangerous due to their role as the main coronary trunk, easily inducing fatal cardiovascular events (6). Existing data indicate that KD-associated giant coronary artery aneurysms (gCAAs) typically develop in children with severe acute vasculitis and inadequate response to intravenous immunoglobulin (IVIG), with most lesions stabilizing or regressing within 1–2 years after acute-phase treatment (5, 7). Persistent, stable giant LMCA aneurysms extending into young adulthood are extremely rare.

Most adult patients with chronic KD-associated LMCA aneurysms reported in the literature present with concomitant myocardial infarction, severe stenosis, or multivessel involvement at initial evaluation (8). In contrast, our patient only manifested mild chest discomfort without acute cardiovascular complications, reflecting an infrequent stable early lesion phenotype. Pathogenically, subclinical medium-vessel vasculitis arising from mild undiagnosed incomplete KD induces focal endothelial injury and elastic fiber breakdown within the LMCA (9). Without timely recognition and intervention, chronic vascular remodeling gradually results in fusiform aneurysmal dilatation. Sustained abnormal intracavitary hemodynamics further promote mural calcification and sluggish blood flow, elevating the risks of intralesional thrombosis and secondary stenosis. The female predilection for this rare LMCA phenotype may stem from sex-based disparities in immune inflammatory responses during KD onset (10), which requires validation by larger clinical cohorts.

Giant coronary aneurysms in young adults are commonly misdiagnosed as atherosclerotic lesions or congenital vascular malformations. The primary diagnostic pitfall highlighted by this case is the under-recognition of asymptomatic incomplete KD lacking the classic childhood fever and exanthema. For young adults presenting with unexplained isolated giant coronary aneurysms lacking conventional atherosclerotic risk factors, remote undiagnosed KD sequelae should rank high in the differential diagnosis. Unlike the original draft that overemphasized angiographic findings as diagnostic proof, the revised discussion clarifies that angiographic morphology only serves as supportive evidence, while complete exclusion of all alternative vasculopathies is the prerequisite to establish the presumptive diagnosis of KD sequelae. We further supplement standardized morphological comparisons referenced from two authoritative sources: the 2024 AHA KD Scientific Statement detailing typical KD coronary aneurysm features, and Gordon et al.’s landmark 2009 JACC review summarizing decades of angiographic data on KD-related coronary ectasia. The multifocal beaded fusiform and saccular dilatation seen in our patient is a unique signature of prior KD vasculitis that cannot be replicated by atherosclerosis, large-vessel vasculitis or congenital coronary anomalies, reinforcing the rationality of our diagnostic inference.

This case provides valuable clinical insights for the diagnosis and management of adult KD residual lesions. First, it broadens the diagnostic scope of adult coronary aneurysms: KD sequelae are not limited to pediatric populations, and undiagnosed incomplete KD is a critical occult cause of idiopathic giant coronary aneurysms in young adults. Second, standardized long-term management is mandatory for stable KD-related gCAAs. Even in asymptomatic patients with quiescent inflammation, persistent antithrombotic therapy and regular imaging surveillance are essential to prevent life-threatening thrombotic complications. Third, giant LMCA aneurysms carry substantial cardiovascular risk; individualized dynamic monitoring of aneurysm diameter, mural calcification, and intracavitary hemodynamics is required to identify the optimal timing for percutaneous or surgical revascularization. Sekimoto et al. (11) published a long-term follow-up case of KD-related thrombotic coronary aneurysm complicated by fatal myocardial infarction 35 years after childhood KD onset, matching the decades-long cumulative thrombotic risk timeline of our patient. This paper provides standardized long-term thrombotic risk stratification frameworks for adult KD survivors with persistent giant coronary aneurysms, which guided our selection of lifelong dual antiplatelet therapy combined with warfarin anticoagulation (INR target 2.0–2.5) to mitigate long-term thrombus and MI risk. Both publications reinforce the lifelong cardiovascular hazard of untreated occult KD coronary lesions.

### 3.1 Limitations of the present study

This study has several important limitations that should be acknowledged when interpreting its findings.

1. Retrospective presumptive diagnosis without confirmatory evidence: There are no preserved childhood medical records to objectively verify prior subclinical incomplete KD during early life, and no vascular biopsy or pathological specimens are available to confirm chronic vasculitic sequelae of KD. The diagnosis remains entirely presumptive, only inferred backward from adult coronary angiographic manifestations and thorough exclusion of alternative vasculopathies, with no specific confirmatory biomarkers for remote childhood KD.
2. Limited short-term follow-up: Clinical and angiographic surveillance was restricted to merely 6 months. Thrombotic complications associated with giant coronary aneurysms carry cumulative lifelong risks; the lack of major adverse cardiovascular events over this brief monitoring window cannot prove the long-term safety or sustained efficacy of combined antiplatelet and anticoagulant therapy. Annual multi-modality imaging follow-up for a minimum of 5 years is necessary to reliably evaluate aneurysm remodeling dynamics and long-term thrombotic risk.
3. Absence of serial long-term large-vessel imaging: Although we retrospectively analyzed CCTA covering the thoracic aorta and supra-aortic branches to rule out quiescent Takayasu arteritis, continuous long-term surveillance of systemic large vessels was not performed.
4. Single-case design without cohort validation: This work is a standalone case report with no comparative large-cohort data, so we cannot validate potential sex-based disparities in the progression of giant left main coronary artery aneurysms arising from occult incomplete KD, and the conclusions cannot be broadly generalized to all analogous patients.
5. Lack of intracoronary high-resolution intravascular imaging (IVUS/OCT): Neither intravascular ultrasound (IVUS) nor optical coherence tomography (OCT) was implemented during invasive coronary angiography and percutaneous coronary intervention in this patient. These high-resolution intracoronary imaging modalities could provide valuable supplementary anatomical information, including precise measurement of aneurysm wall thickness, distribution of vascular calcification, microstructural features of atherosclerotic plaques, and subtle intramural dissection lesions. Such detailed morphological information would help further clarify the chronic vascular pathological changes induced by long-term sequelae of presumed childhood Kawasaki disease. Therefore, the absence of IVUS and OCT examinations constitutes an important limitation of the present case report.

## 4 Conclusion

Giant LMCA aneurysm is a rare but life-threatening long-term sequela of undiagnosed incomplete Kawasaki disease, which may manifest in adult females without the typical acute inflammatory symptoms of childhood KD (12). For young adult patients with unexplained isolated giant coronary aneurysms and no traditional atherosclerotic risk factors, occult residual KD-related vascular lesions should be actively screened. Standardized long-term antithrombotic therapy and close cardiovascular imaging surveillance are crucial to prevent fatal complications including intracoronary thrombosis and acute myocardial infarction. This case supplements the limited clinical data regarding KD-associated giant LMCA aneurysms in young female adults, and provides a practical reference for the early identification and standardized management of atypical long-term vascular sequelae secondary to KD. All diagnostic inferences in this manuscript represent presumptive conclusions rather than definitive confirmation, given the absence of gold-standard testing to verify remote childhood incomplete KD and inability to retrospectively meet 2024 AHA pediatric acute-phase incomplete KD scoring criteria.

## Data Availability

The raw data supporting the conclusions of this article will be made available by the authors, without undue reservation.

## References

[1] Jone PN Tremoulet A Choueiter N Dominguez SR Harahsheh AS Mitani Y . Update on diagnosis and management of Kawasaki disease: a scientific statement from the American Heart Association. Circulation. (2024) 150:e481–500. doi: 10.1161/CIR.0000000000001295, 39534969

[2] Tadokoro Y Kainuma S Tsuda E Asaumi Y Kurosaki K Fukushima S. Giant coronary aneurysms after delayed diagnosis of young adult-onset Kawasaki disease. JACC Case Rep. (2025) 30:105861. doi: 10.1016/j.jaccas.2025.105861, 41386953 PMC12861653

[3] Gordon JB Kahn AM Burns JC. When children with Kawasaki disease grow up: myocardial and vascular complications in adulthood. J Am Coll Cardiol. (2009) 54:1911–20. doi: 10.1016/j.jacc.2009.04.102, 19909870 PMC2870533

[4] Murakami D Sugita G Gunduz M Suenaga T Takeuchi T Suzuki H . Adult onset Kawasaki disease presenting with acute epiglottitis findings. Braz J Otorhinolaryngol. (2020) 86:67–71. doi: 10.1016/j.bjorl.2017.09.001, 29102400 PMC9422665

[5] Lee S Harahsheh AS Raghuveer G Portman MA Sabati AA Khoury M . Spectrum of coronary artery involvement with multisystem inflammatory syndrome in children versus Kawasaki disease. J Am Heart Assoc. (2025) 14:e037761. doi: 10.1161/JAHA.124.037761, 40265601 PMC12184260

[6] Shimizu C Pérez Pomares JM Loy CJ de Vlaminck I Tremoulet AH Ruiz-Villalba A . Pathology of the coronary arteries and myocardium in Kawasaki disease. Annu Rev Pathol. (2026) 21:349–71. doi: 10.1146/annurev-pathmechdis-111523-023453, 41150874

[7] Agrafiotou A Sapountzi E Margoni A Fotis L. Immunophenotype of Kawasaki disease: insights into pathogenesis and treatment response. Life (Basel). (2025) 15:1012. doi: 10.3390/life15071012, 40724515 PMC12299537

[8] Onoki T Metoki T Iwasawa S Kawano K Kimura M Kure S . Two distinct cases of adult-onset Kawasaki disease. Intern Med. (2022) 61:3525–9. doi: 10.2169/internalmedicine.9044-21, 35466164 PMC9790799

[9] Goel AR Yalcindag A. An update on Kawasaki disease. Curr Rheumatol Rep. (2024) 27:4. doi: 10.1007/s11926-024-01167-4, 39625646

[10] Mueller F Knirsch W Harpes P Prêtre R Valsangiacomo Buechel E Kretschmar O. Long-term follow-up of acute changes in coronary artery diameter caused by Kawasaki disease: risk factors for development of stenotic lesions. Clin Res Cardiol. (2009) 98:501–7. doi: 10.1007/s00392-009-0032-2, 19499164

[11] Sekimoto T Shiraki T Tanaka T Kawakami R Kutys R Virmani R . Long-term Kawasaki disease complication: thrombotic coronary aneurysm leading to acute myocardial infarction. JACC Case Rep. (2024) 29:102503. doi: 10.1016/j.jaccas.2024.102503, 39359519 PMC11442342

[12] Gehrmann A Morwood K Gillis D Coleman T Subedi S. A case of adult-onset Kawasaki disease. Med J Aust. (2018) 208:250–1. doi: 10.5694/mja17.00949, 29614935

